# The rectus abdominis tendon insertion to the pubic bone and its clinical implications: A cadaveric study

**DOI:** 10.1051/sicotj/2024053

**Published:** 2025-01-20

**Authors:** Evangelos A. Tourvas, Aristidis H. Zibis, Michail E. Klontzas, Apostolos H. Karantanas, Johannes D. Bastian, Theodoros H. Tosounidis

**Affiliations:** 1 Department of Orthopaedic Surgery, Medical School, University of Crete 71110 Heraklion Greece; 2 Department of Anatomy, Faculty of Medicine, University of Thessaly 41334 Larissa Greece; 3 Department of Radiology, Medical School, University of Crete 71110 Heraklion Greece; 4 Department of Orthopaedic Surgery and Traumatology, Inselspital, Bern University Hospital, University of Bern 3010 Bern Switzerland

**Keywords:** Rectus abdominis tendon, Adductor longus tendon, Pubic bone, Anterior intrapelvic approach, Pfannenstiel incision, Cadaveric study

## Abstract

*Purpose*: The primary aim of this study is to determine the rectus abdominis tendon (RAT) insertional anatomy and consequently clarify the extension of secure mobilization of the tendon from the pubic bone in the setting of anterior approaches in pelvic and acetabular reconstruction surgery. *Materials and methods*: Eleven fresh frozen cadaveric pelvises were dissected by two fellowship-trained orthopaedic trauma surgeons utilizing the anterior intrapelvic approach (AIP). The RAT at the pubic body was dissected, and its footprint on the pubic bone was defined, marked, and measured. *Results*: Nineteen (19) RAT insertions were analyzed. The average total medial vertical length was 33 mm (range 26–42 mm), and the average total lateral vertical length was 36.5 mm (range 26–46 mm). The total width of the proximal insertion on both sides was measured at an average of 20.42 mm (range 14–24 mm). The average width of the tendon at the transition area between the cranial and caudal areas of the pubic bone was 16.45 mm (range 12–22 mm). The average distal insertion width of the RAT was less than the proximal and middle widths, measuring 10.45 mm (range 8–13 mm). *Conclusion*: The tendon can be safely mobilized up to an average total medial vertical length of 33 mm (and in no case more than 42 mm) and to an average total lateral vertical length of 36.5 mm (and in no case more than 46 mm). This piece of anatomical information will equip orthopaedic surgeons with a better understanding of the insertional anatomy of the RAT and subsequent safer surgical release when performing anterior approaches to the pelvic ring.

## Introduction

The insertion of the rectus abdominis tendon (RAT) into the pubic bone is of significant interest to orthopedic trauma surgeons, sports medicine surgeons, and radiologists alike. In orthopaedic trauma surgery, the tendon is a crucial anatomic structure in Pfannenstiel and anterior intrapelvic (AIP) approaches utilized for managing pelvic and acetabular fractures. Mobilization of the rectus abdominis muscle insertion tendon of the pubic bone is an integral, essential surgical step of these surgical approaches [1]. The extended mobilization of the RAT has been recommended as a key manoeuvre to facilitate better exposure of the pubic bone and, consequently, better access to the more lateral-sided structures [2]. Complete detachment of the RAT is not recommended [3–5]. To date, the anatomy of the RAT insertion has not been studied in full detail, and the extension of safe distal and lateral mobilization/detachment is unknown. The primary aim of this study is to determine the RAT insertional anatomy and to clarify the extension of secure mobilization of the tendon from the pubic bone in the setting of anterior approaches in pelvic and acetabular reconstruction surgery.

Moreover, RAT is involved in sports-related groin pain [6–8] and is an anatomical area of interest for sports medicine surgeons and radiologists. It has been proposed that the opposing forces from the rectus and adductor longus muscle lead to lesions described as the so-called “sports hernia” [6–10]. A detailed knowledge of the adductor longus insertion to the pubic bone could assist in safely guiding the specialist physician treating pubalgia when performing diagnostic or therapeutic procedures. The secondary aim of this study is to determine the adductor longus tendon (ALT) insertion in relation to the RAT insertion.

To the best of our knowledge, this is the first study in the contemporary orthopaedic literature that is trying to provide relevant clinical information on the insertional anatomy of RAT and ALT.

## Materials and methods

Eleven fresh frozen cadaveric pelvises (6 males and 5 females) were dissected by two fellowship-trained orthopaedic trauma surgeons utilizing the anterior intrapelvic approach. Any specimens that had undergone prior pelvic, urogenital, and hernia repair surgery were excluded from the study. Two of the specimens had evidence of inguinal hernia surgery on the left side, and for that reason, this side of each cadaver was excluded from our measurements. In total 19, hemipelvices with 19 RAT insertions were included and analyzed in this study.

**Figure 1:**
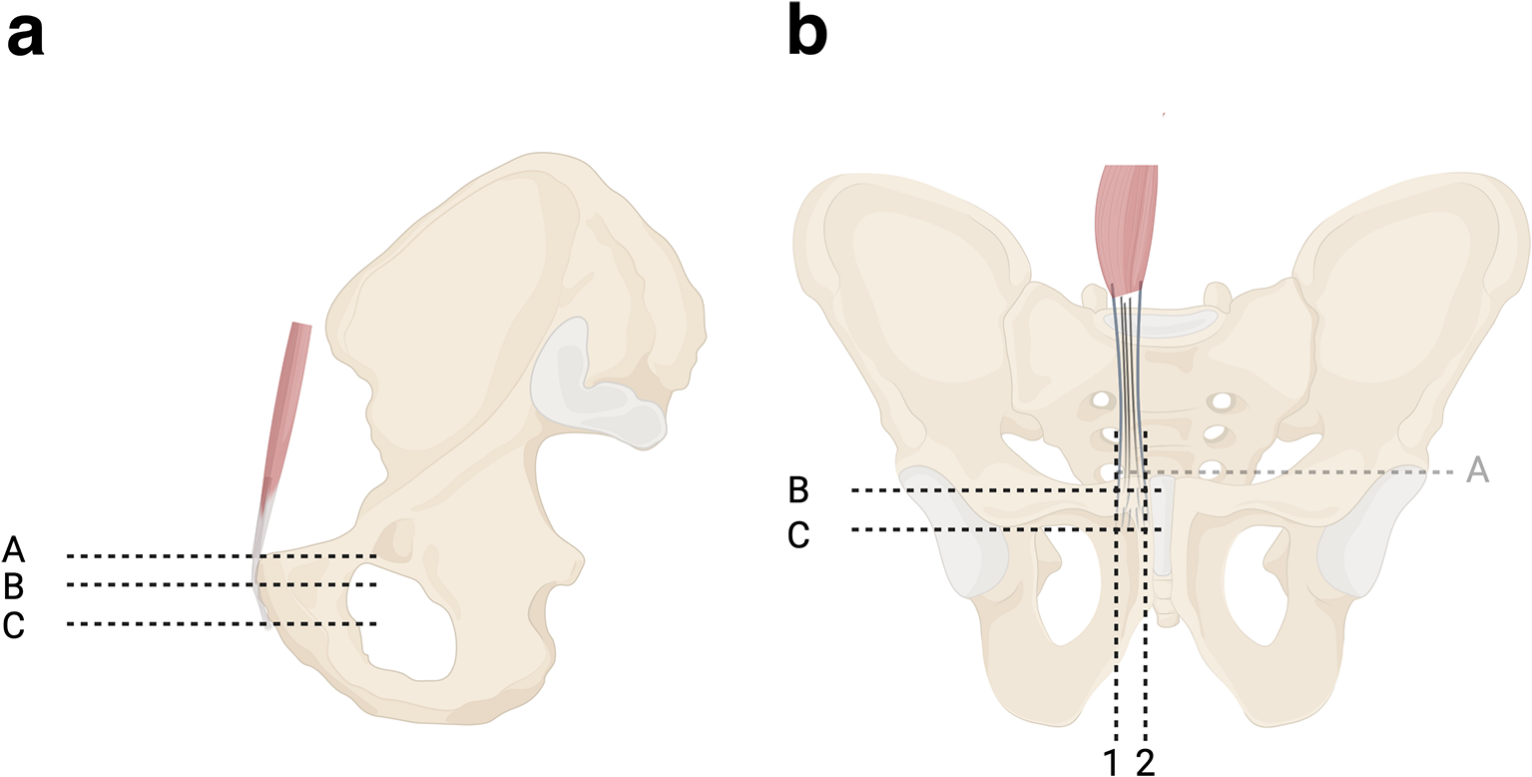
Schematic drawing showing the two parts of the insertion of RAT to the pubic bone at the sagittal (a) and coronal planes (b). A-B cranial part, B-C caudal part.

**Figure 2:**
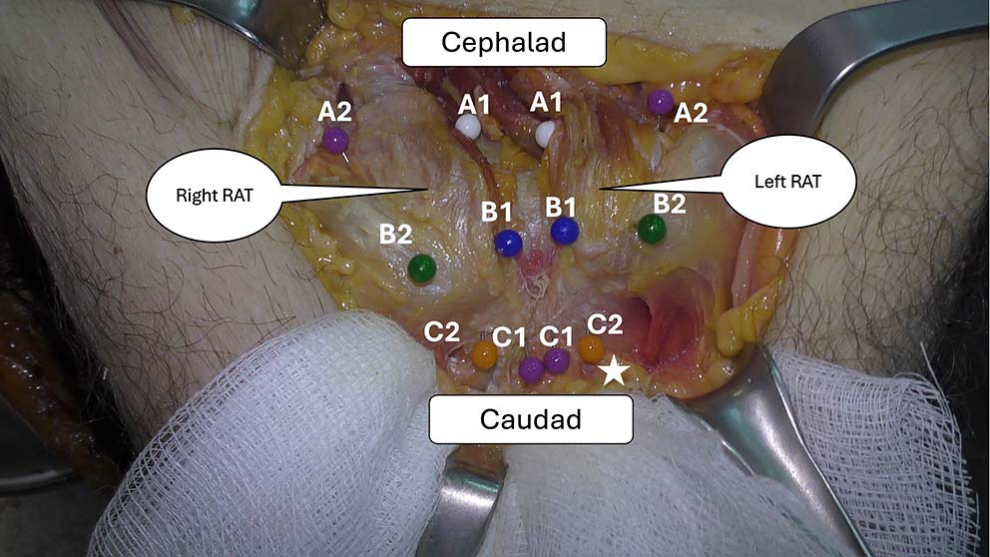
Anatomic dissection showing the proximal, transitional, and distal insertion of the RAT to the pubic bone. A1 and A2: proximal medial and lateral insertions. B1 and B2: transitional medial and lateral insertions. C1 and C2: distal medial and lateral insertions. Asterisk: the insertion of ALT.

**Figure 3:**
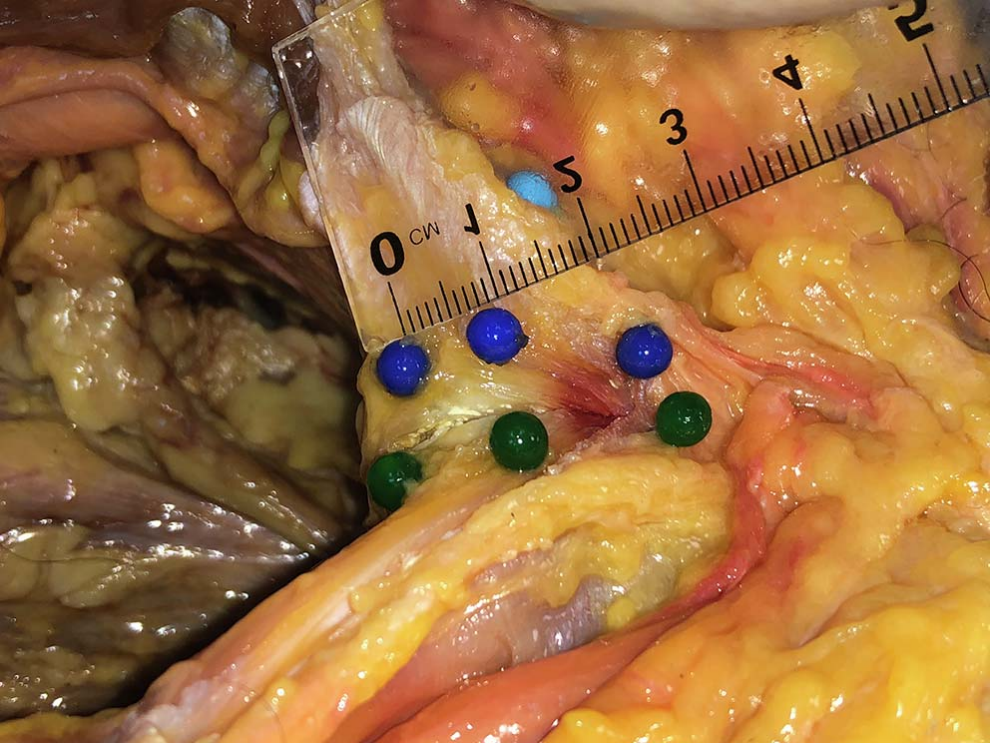
Anatomic dissection of the left RAT (blue pins) demonstrates its insertion as seen from the right side when the surgeon performs its detachment from the pubic bone (A1–B1 cranial part, B2–C2 caudal part). Green pins depict the insertion of the right RAT.

**Figure 4:**
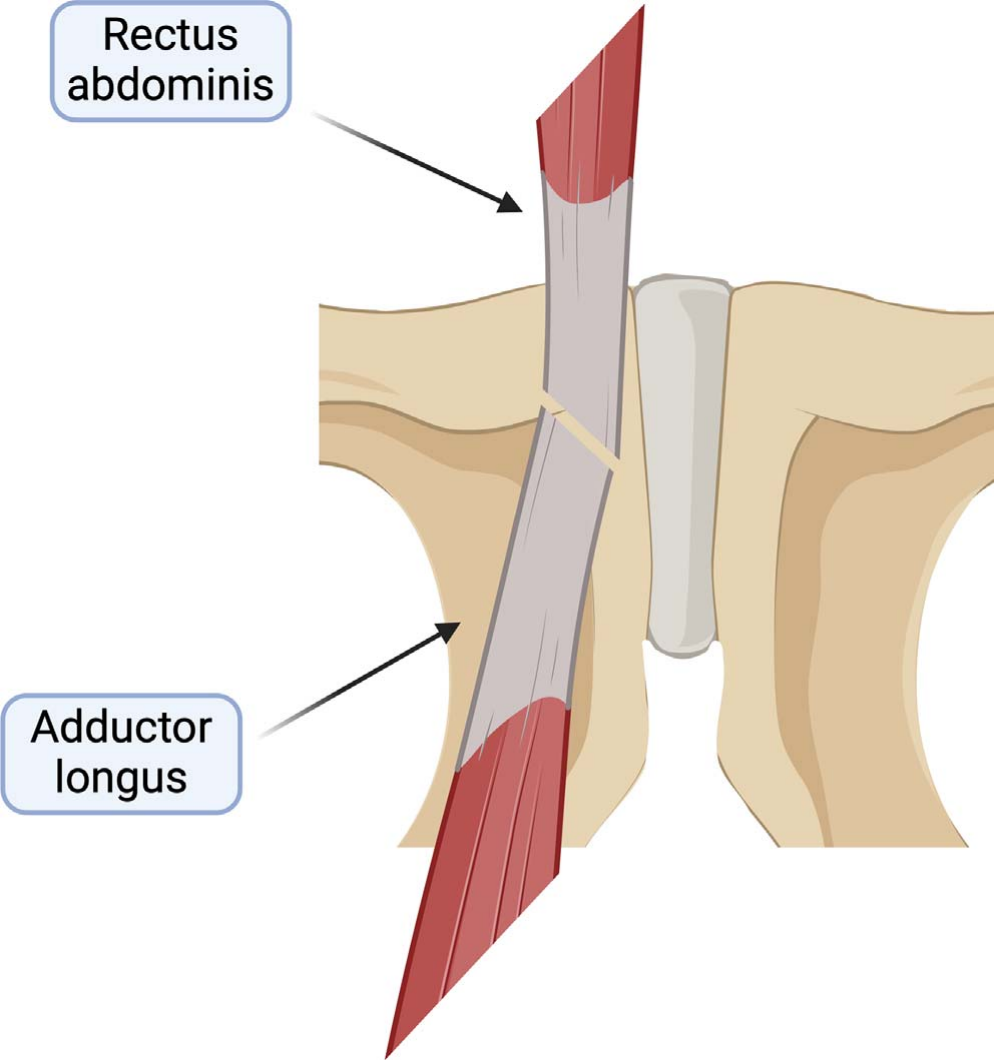
Schematic drawing demonstrating the relationship of the RAT and ALT.

A 10–12 cm transverse (Pfannenstiel) incision, 3–4 cm proximal to the pubic symphysis, was performed. The Linea Alba was identified and dissected, and the left and right RAT insertions were identified and meticulously mobilized from the surrounding tissues. We aimed to measure the vertical and horizontal borders of the tendon at its insertion into the pubic bone. To establish reliable and reproducible measurements, the pubic body was described as having two surfaces, the cranial and the caudal, that create an angle between them (Fig. 1). The RAT inserts onto both of these surfaces. For that reason, the rectus abdominis insertion was measured in parts. Six pins were used to mark the periphery of the insertion of the RAT (Figs. 2 and 3). Two pins (medial-A1 and lateral-A2) were placed at the upper border of the RAT insertion at the cranial surface of the pubic bone. Two pins (medial-B1 and lateral-B2) were placed at the transition area between the cranial and the caudal surface of the pubic bone. Two pins (medial-C1 and lateral-C2) were placed at the distal end of the RAT (caudal surface). C1 pin marked the medial border of the pubic bone at the medial end of the RAT insertion. C2 pin marked the distal lateral end of the RAT and the insertion of ALT (Fig. 4). In all dissections, the C2 point, which was considered the insertion point of the ALT, was meticulously identified. All measurements were performed with the use of a precision caliper. The distances between A1 and B1 (medial proximal vertical) and B1–C1 (medial distal vertical), were recorded to calculate the average length of the medial border of the RAT. The distance between A2 and B2 (lateral proximal vertical) and B2–C2 (lateral distal vertical) was recorded in order to calculate the average length of the lateral border of the RAT. The total medial length was calculated as the sum of the distances A1–B1 and B1–C1, and the total lateral length was calculated as the sum of the distances A2–B2 and B2–C2. In addition, the distances between A1 and A2 (proximal horizontal), B1–B2 (middle horizontal) and C1–C2 (distal horizontal) were measured in order to calculate the average width of the RAT at the proximal insertion, the transitional area and the distal end of the tendon respectively.

### Statistical analysis

Statistical analysis was performed using R programming language as implemented in RStudio (R. v 4.03 and RStudio v. 1.3.1093). Descriptive statistics were used to present data from anatomical measurements. Distances were presented as mean (mm) and range (mm). Comparisons of measurements between sides and sexes were performed with the Mann–Whitney *U* test. Correlation analysis between anatomical measurements was performed with the corrplot package using Pearson correlation coefficients. *P*-values less than 0.05 were used to define statistical significance.

## Results

In total, 19 RAT insertions were analyzed. The average distance of the total medial length of the RAT was 32.78 mm (range 31–37 mm) on the left side and 33.18 mm (range 26–42 mm) on the right side. The total lateral length of the RAT was 36.56 mm (range 31–44 mm) on the left and 35.55 mm on the right side (range 26–46 mm). The average total medial border, regardless of the side, was 33 mm (range 26–42), and the average total lateral border, irrespective of the side, was 36.5 mm (range 26–46).

The total width of the proximal insertion (A1–A2) on both sides was measured at an average of 20.42 mm (range 14–24 mm). The average width of the RAT at its proximal insertion on the pubic bone was 20.78 (range 18–24) mm on the left and 20.14 (range 14–24 mm) mm on the right. The average width of the tendon at the transition area between the cranial and caudal areas (B1–B2) of the pubic bone was 16.45 mm (range 12–22 mm), with 16 (range 12–22) mm on the left and 16.82 (range 12–21) mm on the right side. The average insertion of the RAT on its distal end (C1–C2) was shorter than the proximal and the middle width with an average width of the RAT insertion at its distal end of 10.45 (range 8–13) mm, with 9.89 (range 8–11) mm on the left and 10.91 (range 9–13) mm on the right.

No statistically significant differences were found between the right and the left sides (*P* value > 0.05). The only measurements that were found to differ significantly between male and female cadavers were the lateral distal vertical and the total lateral distance (*P* = 0.002 and *P* = 0.013, respectively). Correlation analysis showed that the highest positive correlations (Pearson correlation > 0.7) were found between medial and lateral distal vertical measurements (*r* = 0.74), between medial distal vertical and total medial measurements (*r* = 0.77), between lateral distal vertical and total lateral measurements (*r* = 0.75), as well as between total medial and total lateral measurements. No strong negative correlations (Pearson correlation coefficients < −0.7) were identified between any of the measurements.

## Discussion

To the best of our knowledge, this is the first study in contemporary orthopaedic and anatomic literature that describes the insertion of the RAT into the pubic bone. Our study concludes that the total width on the proximal insertion on both sides was measured at an average of 20.42 mm (range 14–24 mm), the average width of the tendon at the transition area between the cranial and caudal areas (B1–B2) of the pubic bone was 16.45 mm (range 12–22 mm), and the average insertion of the RAT on its distal end (C1–C2) was 10.45 (range 8–13 mm). The distal width of the tendon was significantly narrower than the proximal and middle widths, a fact that indicates that the surgeon should be extremely careful when performing the medial to lateral detachment of the tendon distally. The average total inner length of the RAT on both sides was 33 mm (range 26–42), and the average total lateral length, irrespective of side, was 36.5 mm (range 26–46). According to these measurements, we conclude that it is safe to detach the RAT up to 42 mm at the medial border (A1–C1) and up to 46 mm at the lateral border (A2–C2). The lateral border of the distal tendon is longer than its medial counterpart because the insertion has a trapezoidal shape. This should be taken into account to avoid overzealous dissection and inadvertent distal detachment of the RAT.

In the present study, we also marked the origin of the insertion of the ALT which is the same point as the most distal and lateral insertional point of the RAT (C2). This origin of ALT was identified in all dissections. The results of our study show that the C2 is located 10.45 (range 8–13 mm) (C1–C2) lateral to the inner aspect of the pubic body and 33 (26–42 mm) (A1–C1) distal to the most proximal inner insertion of RAT. These findings also add to a few recent studies that have investigated the musculotendinous attachments at the PS [11] and the ALT-origin anatomy [12].

The limitations of this study include the relatively small number of cadaveric dissections and the absence of relevant anthropometric data on the cadavers used. Nevertheless, the sample size was similar to the number of cadaveric specimens utilized in similar anatomical studies that investigated insertional areas of other tendons [13–19]. In light of the findings of the present study, we are currently conducting an MR imaging study, with a higher sample size, which we hope will further improve our knowledge of the insertional anatomy of the RAT and ALT.

The management of pelvic and acetabular fractures necessitates an advanced level of surgical anatomy knowledge and specific surgical skills [20]. The AIP approach is one of the most frequently used approaches for the surgical treatment of these fractures and allows excellent visualization for fracture reduction and fixation [21–23]. The AIP is also utilized for the surgical management of symptomatic hip dysplasia in adolescents and young adults [24]. Mobilization of the rectus abdominis is considered of paramount importance to achieve improved access to the anterior column, quadrilateral surfaces and posterior column of the acetabulum. For that, at the initial steps of the approach, the tendon of the rectus abdominis is carefully detached from the pubic bone (from medial to lateral and from proximal to distal). Hohman retractors are placed at the lateral aspect of the pubic tubercle, and they assist in exposing the cranial and caudal surfaces of the pubic body [1, 3, 4] and allowing access to more lateral structures such as the pectineus muscles insertion to the relevant pubic bone recess and iliopectineal insertion at the iliopectineal eminence. The results of this study can assist the practising surgeon in identifying the secure limits of the RAT mobilization when performing the AIP. This piece of knowledge would prevent accidental complete detachment of the tendon from the pubic, a complication that can be difficult to deal with and might potentially have devastating consequences to the patient, such as the early or late development of an iatrogenic direct inguinal hernia and insufficiency of the anterior abdominal musculature. Our study is the first one in contemporary literature to provide data about the anatomy of the insertion of the RAT, and consequently, it should be considered a baseline study for future investigations. Additionally, the herein work identified the adductor longus tendon (ALT) insertion in relation to the RAT insertion, which is a piece of knowledge that can guide safe and accurate diagnostic and therapeutic injections in the area.

## Conclusion

The average total medial and lateral lengths of the RAT are 33 mm and 36.5 mm, respectively. The maximum total medial and lateral lengths of the RAT were measured at 42 mm and 46 mm, respectively. Additionally, the width of the most distal part of the RAT was measured at 10.45 mm and in no case wider than 13 mm.

This piece of anatomical knowledge provides surgeons with a better understanding of the footprint anatomy when performing the anterior intrapelvic approach.

The origin of ALT is located 10.45 (range 8–13 mm) lateral to the inner aspect of the pubic body and 33 (26–42 mm) (A1–C1) distal to the most proximal inner insertion of RAT. This information could be helpful to physicians who perform diagnostic and therapeutic injections in the area.

## Data Availability

Data are not publicly available but is available on request, due to privacy/ethical restrictions.
